# Elucidation of composition of chlorine compounds in acidic sodium chlorite solution using ion chromatography

**DOI:** 10.1371/journal.pone.0289534

**Published:** 2023-08-10

**Authors:** Ayuta Kishimoto, Ryosuke Ohtsubo, Yuta Okada, Kenta Sugiyama, Hisataka Goda, Toshikazu Yoshikawa, Masahiro Kohno, Koji Fukui

**Affiliations:** 1 Department of Bioscience and Engineering, Molecular Cell Biology Laboratory, College of System Engineering and Science, Shibaura Institute of Technology, Fukasaku, Minuma-ku, Saitama, Japan; 2 Department of Systems Engineering and Science, Molecular Cell Biology Laboratory, Shibaura Institute of Technology, Graduate School of Engineering and Science, Fukasaku, Minuma-ku, Saitama, Japan; 3 Sankei Co. Ltd., Shiromi, Chuo-Ku, Osaka, Japan; 4 Louis Pasteur Center for Medical Research, Tanaka Monzen-cho, Sakyo-ku, Kyoto, Japan; 5 Kyoto Prefectural University of Medicine, Kawaramachi-Hirokoji, Kajii-cho, Kamigyo-ku, Kyoto, Japan; University of New England Faculty of Arts and Sciences: University of New England, AUSTRALIA

## Abstract

With the spread of coronavirus infections, the demand for disinfectants, such as a sodium chlorite solution, has increased worldwide. Sodium chlorite solution is a food additive and is used in a wide range of applications. There is evidence that chlorous acid or sodium chlorite is effective against various bacteria, but the actual mechanism is not well understood. One reason for this is that the composition of chlorine-based compounds contained in sodium chlorite solutions has not been clearly elucidated. The composition can vary greatly with pH. In addition, the conventional iodometric titration method, the *N*,*N*-diethyl-*p*-phenylenediamine sulfate (DPD) method and the absorption photometric method cannot clarify the composition. In this study, we attempted to elucidate the composition of a sodium chlorite solution using absorption spectrophotometry and ion chromatography (IC). IC is excellent for qualitative and quantitative analysis of trace ions. Through this, we aimed to develop an evaluation method that allows anyone to easily determine the bactericidal power of sodium chlorite. We found that commercially available sodium chlorite solution is 80% pure, with the remaining 20% potentially containing sodium hypochlorite solution. In addition, when sodium chlorite solution became acidified, its absorption spectrum exhibited a peak at 365 nm. Sodium chlorite solution is normally alkaline, and it cannot be measured by the DPD method, which is only applicable under acidic conditions. The presence of a peak at 365 nm indicates that the acidic sodium chlorite solution contains species with oxidizing power. On the other hand, the IC analysis showed a gradual decrease in chlorite ions in the acidic sodium chlorite solution. These results indicate that chlorite ions may not react with this DPD reagent, and other oxidizing species may be present in the acidic sodium chlorite solution. In summary, when a sodium chlorite solution becomes acidic, chlorine-based oxidizing species produce an absorption peak at 365 nm. Sodium hypochlorite and sodium chlorite solutions have completely different IC peak profiles. Although there are still many problems to be solved, we believe that the use of IC will facilitate the elucidation of the composition of sodium chlorite solution and its sterilization mechanism.

## Introduction

The coronavirus disease 2019 (COVID-19) pandemic changed the world [1,2]. The rapid spread of the virus has transformed our lives, and the use of disinfection has become part of our daily routine [3]. Ethanol is the most commonly used disinfectant [4]. However, during the rapid spread of the infection, there was an ethanol shortage, and it became a societal problem in terms of resale markups and hoarding. Sodium hypochlorite (NaOCl) has been commonly used to replace the scarce ethanol. Sodium hypochlorite solution is a chlorine-based disinfectant and is also commonly used as a bleaching agent [5,6]. However, it is very dangerous to the human body because chlorine gas is generated if it is used incorrectly [7]. For this reason, a disinfectant is required that is safe for the human body and has sterilizing power against viruses and bacteria.

One candidate among chlorinated compounds is chlorous acid solution [8]. Chlorous acid (HClO_2_) has a structure with one more oxygen than hypochlorous acid. Chlorous acid solution is also approved by the Ministry of Health, Labour and Welfare of Japan for use as a food additive and is often used for washing red meat, seafood, and raw vegetables in factories [9–11]. Much evidence has demonstrated that a weakly acidic (pH 5.0–6.0) sodium chlorite solution has a stronger disinfectant effect than the alkaline version [12,13]. Thus, chlorite-related solutions are relatively safe for the human body despite their disinfecting power. It is believed that chlorous acid solution contains three kinds of chlorine-based compounds: chlorous acid (HClO_2_), chlorite ions (ClO_2_^-^) and chlorine dioxide (ClO_2_) [12]. However, just as the bactericidal power varies greatly depending on the pH, the composition may strongly depend on the pH and storage conditions. In other words, the details of the types and amounts of chlorine compounds included in these solutions are unknown.

To identify the compounds, many measurement methods have been developed and used to date. Absorption photometry, iodometric titration (KI), the *N*,*N*-diethyl-*p*-phenylenediamine sulfate (DPD) method, and electron spin resonance (ESR) are usually used to identify and quantify chlorine compounds contained in chlorous acid solution [14,15]. The results obtained are also used as indicators of bactericidal activity [11]. Total chlorine can be calculated by the KI method, and free available chlorine (FAC) can be calculated by the DPD method [15,16]. However, it is not clear what species in the sodium chlorite solution possess disinfecting action. If they can be identified, we will be able to manufacture low-cost, low-dose, long-term-storage disinfectants.

Ion chromatography (IC) is a type of liquid chromatography that can qualitatively and quantitatively identify ions in a solution [17]. Multiple types of ions can be analyzed simultaneously in one analysis, and ions with different participation numbers and valences can be detected separately. In addition, since analysis is performed almost automatically using an autosampler, it has the advantage of being highly reproducible and not dependent on individual skills. IC is commonly used, for example, to measure residual inorganic anion and cation concentrations in river water and wastewater [18,19]. We investigated whether IC could be used to analyze solutions in which multiple chlorinated compounds were mixed. In this study, we attempted to clarify the composition of chlorinated compounds in sodium chlorite solutions under various conditions using ultraviolet-visible (UV/Vis) spectrophotometry, the DPD method and IC.

## Materials and methods

### Preparation of sodium hypochlorite, sodium chlorite and mixture of sodium chlorite and sulfuric acid solutions

6.25, 8, 12, 16, and 32 mM sodium hypochlorite (#197–02206, FUJIFILM Wako Pure Chemical Corp., Osaka, Japan) and same concentrations of sodium chlorite (#014265, FUJIFILM Wako Pure Chemical Corp.) solutions were prepared. To clarify the composition of chlorine compounds in sodium hypochlorite and sodium chlorite solutions, both 8, 16 and 32 mM solutions were diluted 1000-fold and used for IC analysis. A 12.5 mM sodium chlorite solution was mixed with four different concentrations of sulfuric acid. In the IC experiment, each solution was diluted 1000-fold, while in the DPD study, the mixture was diluted 10-, 20- and 50-fold by ultrapure water. All solutions, except for those used for each standard curve, were incubated for 100 min before analysis.

### Observation of maximum absorption wavelength for sodium chlorite solution

Continuous absorption wavelength spectra of sodium chlorite and sodium hypochlorite solutions were measured from 200 to 500 nm using a UV/Vis Microscope and Cuvette Spectrophotometric detector (Multiskan GO, Thermo Fisher Scientific K.K., Tokyo, Japan). Continuous measurement from 200 to 1000 nm was possible with this equipment with an optical path length of 1 nm.

### Creation of standard curves

For analysis of the composition of chlorine compounds in sodium chlorite solution, some standard curves were made. Anion Mixture Standard Solution 1 (including chloride ions) (#019–24011), Chlorate Ion Standard Solution (ClO_3_^-^ 1000, #031–24953), Chlorite Ion Standard Solution (ClO_2_^-^, #036–25581), and Sodium Hypochlorite Solution were used to make each standard curve. Detailed sample information is given in the figure captions. These solutions were purchased from FUJIFILM Wako Pure Chemical Corp.

### Ion chromatograph assay

Some chloride ions, such as chloride (Cl^-^), chlorite (ClO_2_^-^), and chlorate (ClO_3_^-^), were measured by IC (#IC-8100EX, Tosoh Corp., Tokyo, Japan) equipped with suppressed conductivity detection. In this study, a UV detector (#UV-8100, Tosoh Corp.) was added as an optional unit, and set at 365 nm. TSKgel SuperIC-anion HS column (#0022766, for high-throughput analysis, Tosoh Corp.) was used in all analyses, with a column temperature of 40°C. The eluent composition was 7.5 mM sodium hydrogen carbonate and 0.8 mM sodium carbonate, with a flow rate of 1.5 mL/min. Each injection volume was 30 μL, and the detection sensitivity was 500 μS/cm.

### Measurement of free available chlorine volume of sodium chlorite solution

9.5 mL of sodium chlorite solution or each dilution and concentration of the mixture was weighed out and 0.5 mL of pH 6.5 buffer solution was added. Before weighing, the mixture was diluted to three different concentrations and 0.1 g of DPD reagent was added and mixed. The sample was added to the cuvette, and the absorbance at a wavelength of 510 nm was immediately measured using the Multiskan GO.

## Results

### UV/Vis absorption spectra for sodium chlorite and sodium hypochlorite solutions

**Fig 1:**
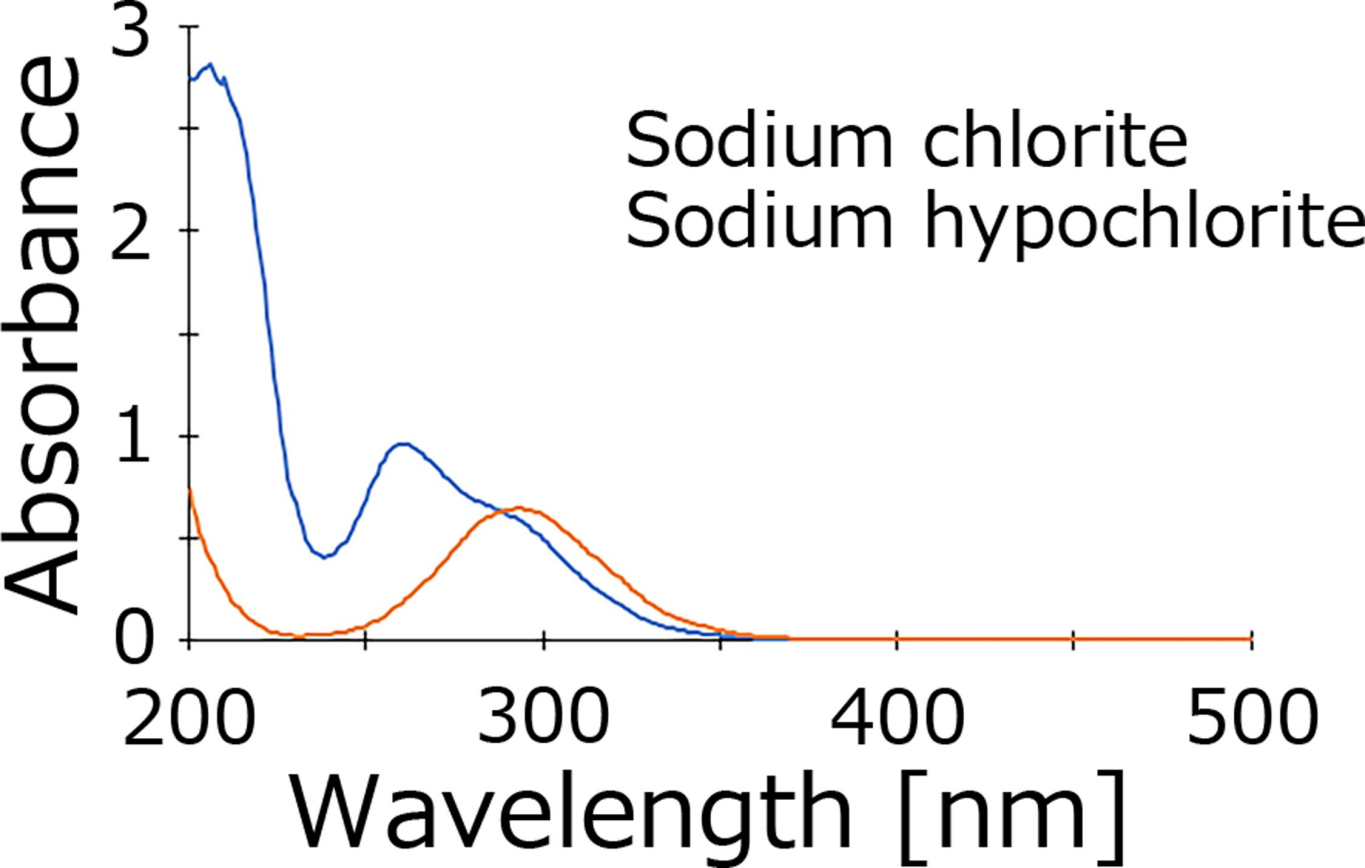
Absorption spectra of sodium chlorite and sodium hypochlorite solutions. 6.25 mM solutions of sodium chlorite (blue) and sodium hypochlorite (orange) were used, and the absorbance was measured from 200 to 500 nm. Each solution was incubated for 100 min before analysis, and at least three independent experiments were performed.

To characterize sodium chlorite and sodium hypochlorite, absorption spectra from 200 to 500 nm were obtained using the Multiskan GO (Fig 1). The maximum absorption wavelength for sodium hypochlorite was 292 nm, and the spectrum contained three absorption peaks at 210, 256 and 292 nm.

### Analysis of sodium chlorite solution using ion chromatography

**Fig 2:**
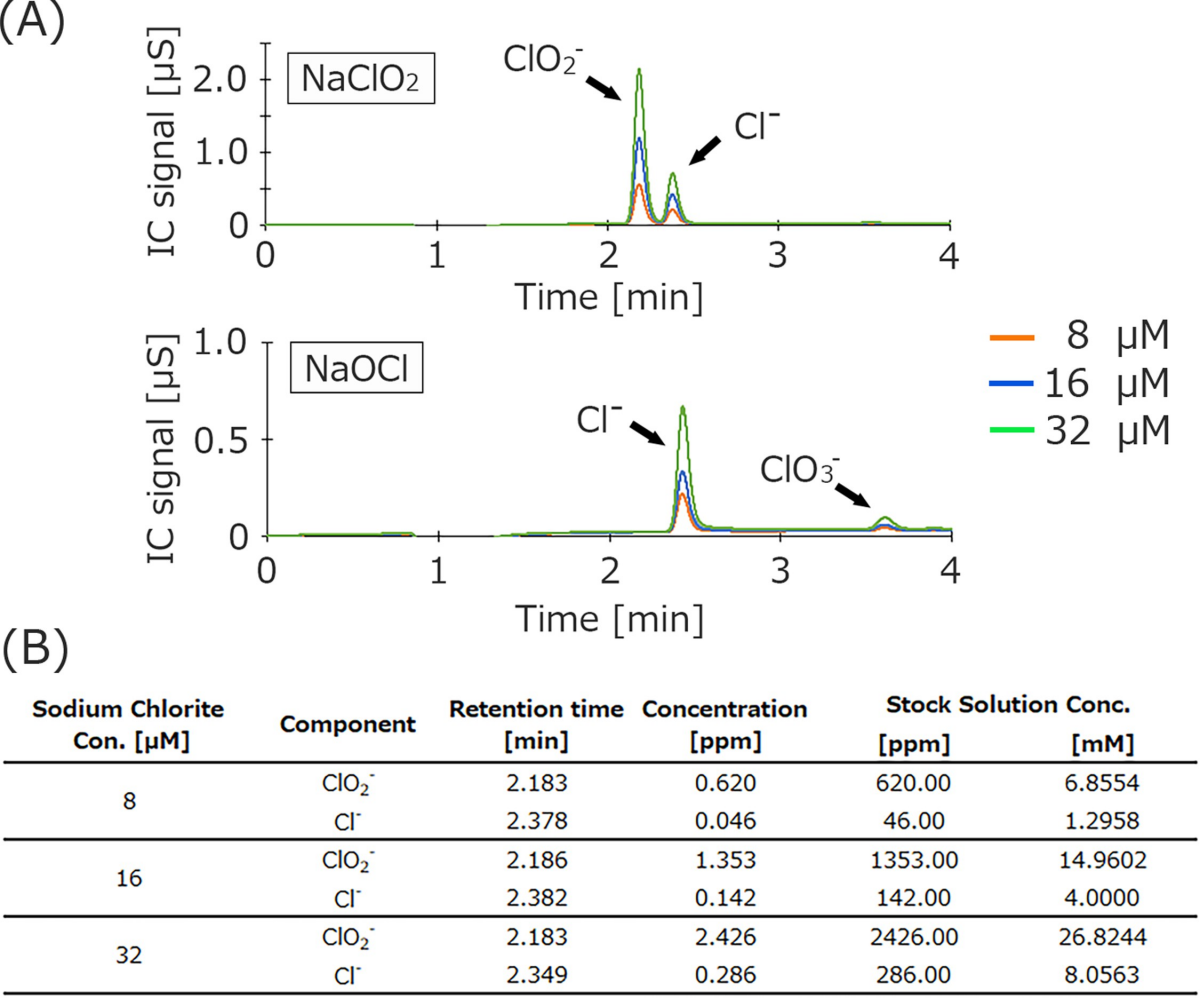
Analysis of three different concentrations of sodium chlorite and sodium hypochlorite solutions using IC. Final concentrations were 8 (orange), 16 (blue) and 32 (green) μM (A). A TSKgel SuperIC-Anion HS column and suppressed conductivity detection were used for the analysis. The eluent was 7.5 mM sodium hydrogen carbonate and 0.8 mM sodium carbonate. The flow rate was 1.5 mL/min. The column temperature was 40°C. The injection volume was 30 μL. The detection sensitivity was 500 μS/cm. After finishing IC, the ion concentration associated with the two peaks for sodium chlorite solution was calculated (B). Each solution was incubated for 100 min before analysis, and at least three independent experiments were performed.

Given that there were three absorption peaks present, three different concentrations of sodium chlorite and sodium hypochlorite solutions were subjected to IC analysis (Fig 2A). Before starting the analysis, three different standard curves were made for chlorite ions (ClO_2_^-^), chloride ions (Cl^-^) and chlorate ions (ClO_3_^-^). The area of each IC peak varied with concentration (data not shown). Three different concentrations of sodium chlorite and sodium hypochlorite solutions were prepared (8, 16 and 32 mM), and before injection each solution was diluted 1,000 times by ultrapure water (for final concentrations of 8, 16 and 32 μM). The analysis of sodium chlorite detected two peaks associated with chlorite and chloride ions. There were also two peaks associated with chloride and chlorate ions for sodium hypochlorite solutions with different concentrations. The calculated ion concentrations in the sodium chlorite solutions are given in Fig 2B.

### Dependence of sodium chlorite absorption spectrum and pH on sulfuric acid concentration

**Fig 3:**
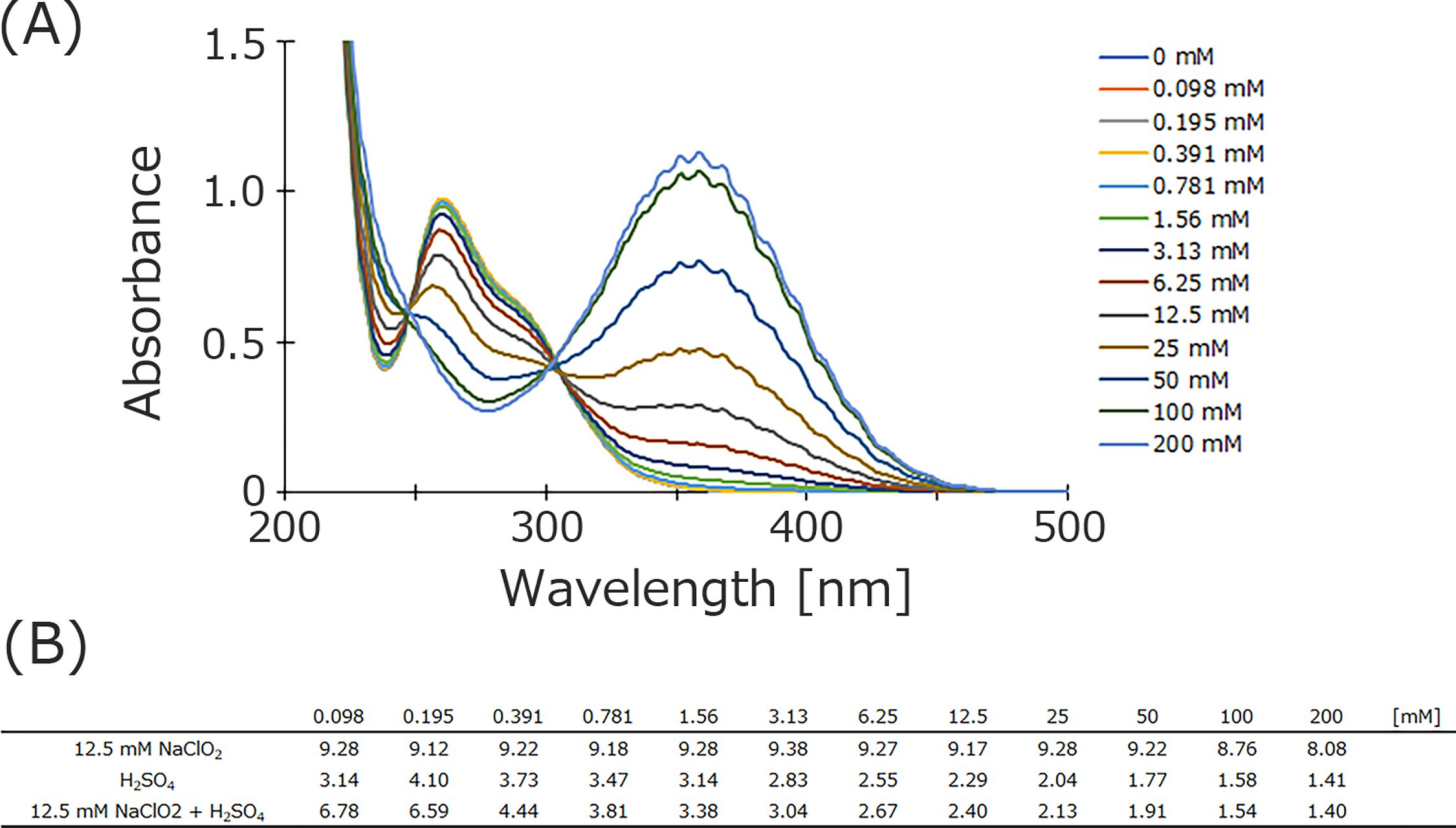
Absorption spectra of 12.5 mM sodium chlorite solutions with different concentrations of sulfuric acid. Measured absorption spectra from 200 to 500 nm (A). pH values (B). Each solution was incubated for 100 min before analysis, and at least three independent experiments were performed.

Sodium chlorite solutions are usually alkaline and exhibit low bactericidal activity. Therefore, acidified sodium chlorite solution (ASC) is usually used [9]. To clarify the composition of chlorine-based compounds when producing a 12.5 mM sodium chlorite solution, the same volume of three different concentrations of sulfuric acid was added to create an acidic condition, followed by analysis using UV/Vis spectrophotometry and pH measurements. With increasing sulfuric acid concentration, the intensity of the absorption peak at 365 nm increased, while that of the peaks at 256 nm and 292 nm decreased (Fig 3A). The pH values are shown in Fig 3B. The mixed solution was made gradually acidic with increasing sulfuric acid concentration.

### Changes in free available chloride volume in acidic sodium chlorite solution

**Fig 4:**
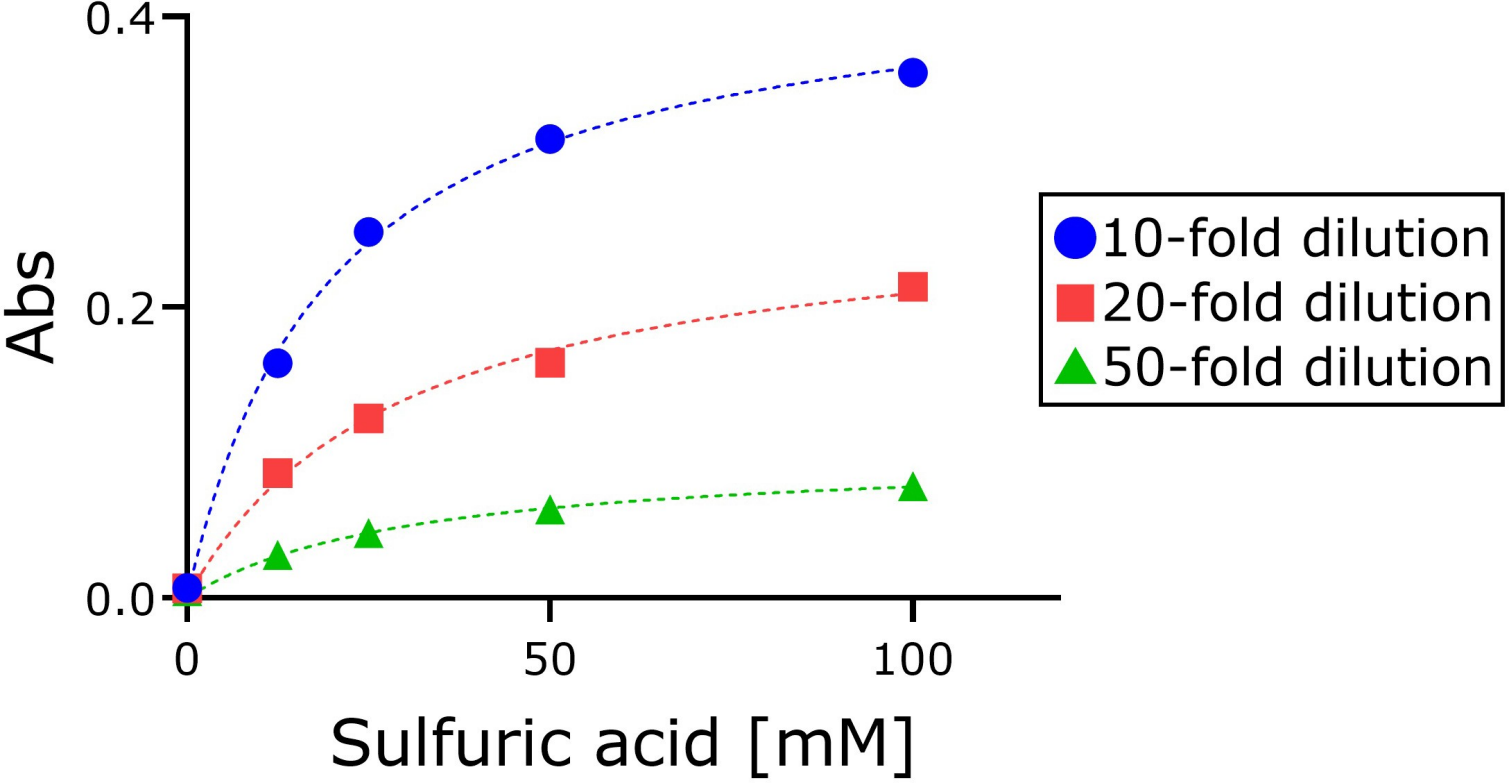
Dependence of absorbance at 510 nm associated with free available chloride on sulfuric acid concentration in sodium chlorite solution. 4.725 mL of 12.5 mM sodium chlorite and the same volume of four different concentrations of sulfuric acid (12.5, 25, 50, and 100 mM) were mixed and incubated for 100 min. Three different concentrations of mixed solution were made by adding ultrapure water and 0.5 mL of buffer solution. 0.1 g DPD reagent was added and the absorbance at 510 nm was measured. At least three independent experiments were performed.

To determine changes in the volume of oxidizing species in acidic sodium chlorite solution, the free available chloride volume was measured by the DPD method (Fig 4). 12.5 mM sodium chlorite and the same volume of four different concentrations of sulfuric acid were added. After 100 min incubation, each mixture was diluted to three different concentrations (10-, 20- and 50-fold) by ultrapure water, the DPD reagent was added, and the absorbance at 510 nm was measured. The free available chloride volume varied with both sulfuric acid concentration and dilution level. No absorbance was detected at 510 nm in the absence of sulfuric acid.

### Dependence of IC peak intensity and pH on sulfuric acid concentration

**Fig 5:**
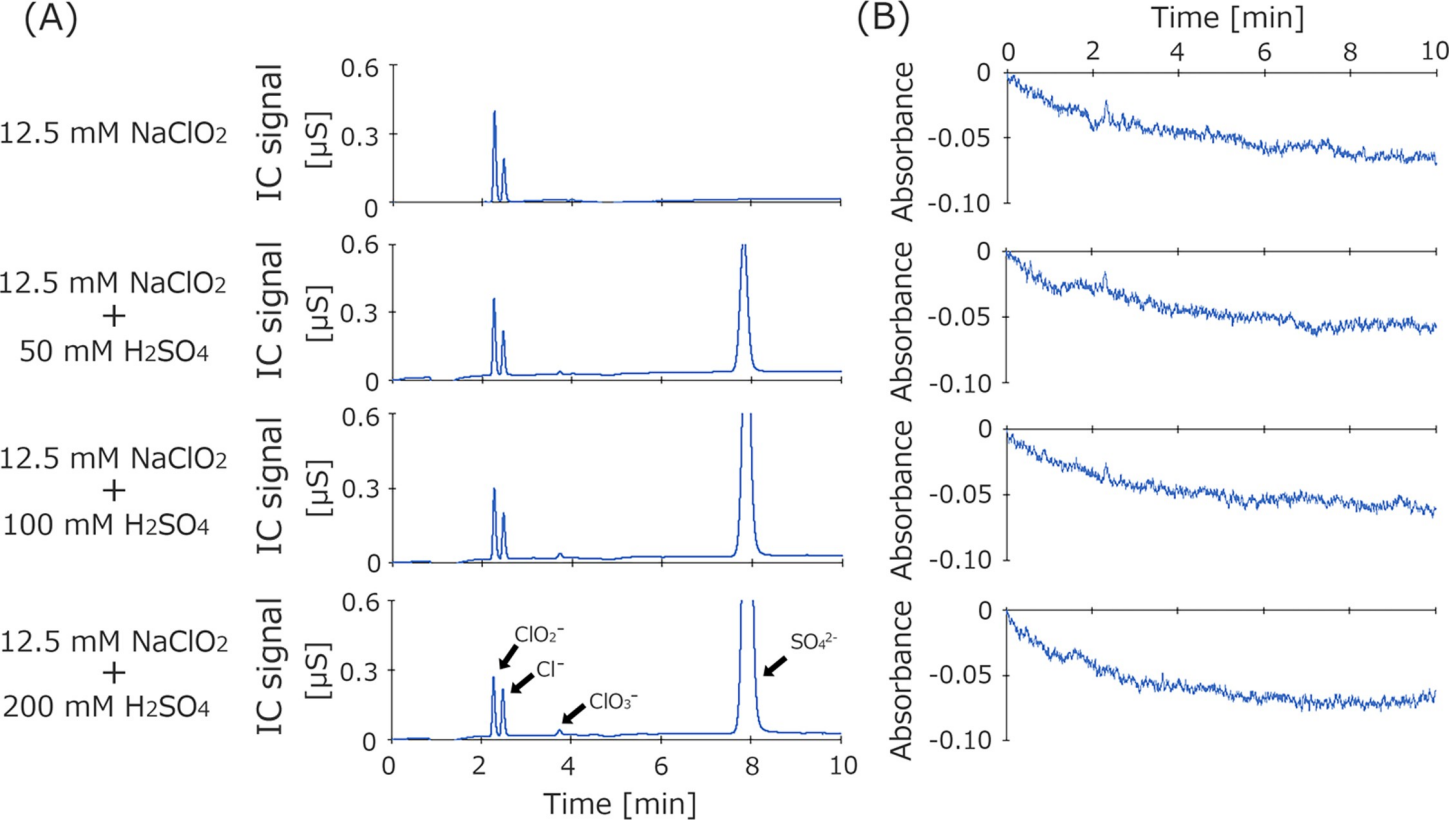
IC analysis of sodium chlorite solutions with different concentrations of sulfuric acid. IC peaks (A). Change in absorbance at 365 nm (B). After absorption measurements (see Fig 3), part of each mixture was diluted 1000 times and 30 μL was injected subjected to IC analysis (final concentration of 12.5 μM NaClO_2_, 12.5 μM NaClO_2_ + 50 μM H_2_SO_4_, 12.5 μM NaClO_2_ + 100 μM H_2_SO_4_, 12.5 μM NaClO_2_ + 200 μM H_2_SO_4_). The eluent was 7.5 mM sodium hydrogen carbonate and 0.8 mM sodium carbonate. The flow rate was 1.5 mL/min. The column temperature was 40°C. The injection volume was 30 μL. The detection sensitivity was 500 μS/cm. At least three independent experiments were performed.

To determine the chloride-related content in mixtures of 12.5 mM sodium chlorite with different concentrations of sulfuric acid, the solutions used for absorption measurements were diluted 1000 times and subjected to IC analysis (Fig 5A). The intensity of the chloride ion peak did not change, but the intensity of the chlorite ion peak gradually decreased with increasing concentration of added sulfuric acid. Chlorate ions were detected at sulfuric acid concentrations of more than 100 mM. No peak at 365 nm was identified by the UV detector after the conductivity detector (Fig 5B).

## Discussion

### The remaining 20% of the 80% pure sodium chlorite solution may be the same as the main component of the sodium hypochlorite solution

The content of chlorinated compounds in different sodium chlorite solutions were determined by spectrophotometry, DPD and IC. A commercially available 80% pure sodium chlorite standard solution was used in this study. The contents of the remaining 20% were not stated on the reagent bottle. In the absorption spectra, three different peaks were detected at 210, 256 and 292 nm (Fig 1). At the same concentration of sodium hypochlorite solution, only the absorption peak at 292 nm was detected. Thus, the unknown species with a maximum absorption wavelength at 292 nm contained in both the sodium chlorite and sodium hypochlorite solutions may be the same.

To further clarify the composition of the species contained in the sodium chlorite solution, IC was used (Fig 2). Sodium chlorite solutions with three different concentrations were prepared and the same two peaks were detected for all samples (at about 2.1 and 2.3 min). Two peaks were also detected for the sodium hypochlorite solution (at about 2.4 and 3.6 min). Before starting the IC analysis, standard solutions containing chloride ions (Cl^-^), chlorite ions (ClO_2_^-^), and chlorate ions (ClO_3_^-^) were prepared. Unfortunately, IC cannot detect hypochlorite ions for unknown reasons. In this study, the retention times for the two detected peaks in the sodium chlorite solution matched those for chlorite and chloride ions, while the retention times for the sodium hypochlorite solution matched those for chloride and chlorate ions. Furthermore, the ratio of chlorite to chloride ions in the sodium chlorite solution was approximately 4 to 1, which is consistent with the 80% chlorite ion purity stated on the reagent bottle. For the sodium hypochlorite solution, the main chloride ion peak intensity increased with increasing concentration. These results suggest that the ions detected at 2.3 min by IC analysis may be the remaining 20% of the species contained in the sodium chlorite solution. In addition, the species may be the same as the main component of the sodium hypochlorite solution.

### The remaining 20% of the 80% pure sodium chlorite could be sodium hypochlorite

As described above, the absorption results suggested that the remaining 20% of the sodium chlorite solution could be the same species as the main component of the sodium hypochlorite solution. Although it is possible that the species corresponding to the 20% chloride ions detected by IC corresponds to the main component of the sodium hypochlorite solution, this seems highly unlikely. The chloride ions detected in the IC analysis may therefore originate from sodium hypochlorite or hypochlorous acid. Sodium hypochlorite solution mainly contains sodium hypochlorite, hypochlorous acid and hypochlorite ions [11,20]. The ratio of sodium hypochlorite to hypochlorous acid changes with pH. At a pH of 4–7, the solution contains mainly hypochlorous acid, whereas for pH >9, it contains mainly sodium hypochlorite with a small amount of hypochlorite ions [21]. Since a sodium hypochlorite solution is usually alkaline, it is considered that the amount of hypochlorous acid is small. Under our experimental conditions, sodium chlorite and sodium hypochlorite solutions were alkaline because they were not mixed with acidic reagents. Even if the ratio changes, the combined amount of sodium hypochlorite and hypochlorous acid would not change. Chloride ions could be produced by both sodium hypochlorite and hypochlorous acid. As a result, the total amount of chloride ions may not change. Therefore, assuming that the remaining 20% of the sodium chlorite solution is sodium hypochlorite, it is thought that there will be no change in the amount of chloride ions detected by IC. The species with the absorption maximum at 292 nm for the sodium hypochlorite solution may be sodium hypochlorite. It is highly unlikely that other unknown chlorinated ions are produced with peaks at the same retention time as chloride ions. If this theory is correct, it would explain the purity problem. However, there are too many unknowns to resolve this issue and further experimentation is required. In any case, sodium chlorite solution (which is about 80% pure) may contain about 20% sodium hypochlorite solution.

### Species with maximum absorption wavelength at 365 nm increased in acidic sodium chlorite solution

Generally, the disinfecting efficacy of sodium chlorite solutions increases gradually as conditions change from alkaline to acidic [13,22]. This tendency suggests that sodium chlorite solution may have a different ion composition under alkaline and acidic conditions. To clarify this, we added different concentrations of sulfuric acid to sodium chlorite solution and measured absorption spectra from 200 to 500 nm using UV/Vis spectrophotometry (Fig 3). Sulfuric acid was chosen over hydrochlorous acid because one of the purposes of this study is to determine the chlorinated compounds in sodium chlorite solutions.

In the absence of sulfuric acid, three major absorption peaks appear at 210, 256 and 292 nm (due to the high intensity of the peak at 210 nm, its top is cut off in Fig 3). The intensity of the peaks at 256 and 292 nm gradually decreased upon sulfuric acid treatment, but that of the peak at 365 nm increased with increasing sulfuric acid concentration. It is unknown what the 210 nm peak corresponds to, but the 256 nm peak is due to sodium chlorite [14]. However, there was no isosbestic point between sodium chlorite solution (256 nm) and sodium hypochlorite solution (292 nm) in the measured absorption spectra. This indicates a low probability that sodium chlorite changes to sodium hypochlorite with changing pH. Rather, the intensity of the peaks for both sodium chlorite and sodium hypochlorite solutions gradually decreased with increasing acidity. Conversely, the intensity of the peak at 365 nm increased as the solutions became more acidic. This indicates the possibility that sodium chlorite or chlorous acid itself is directly transformed into a species that exhibits maximum absorption at a wavelength of 365 nm. However, no peak appeared at 365 nm when only the sodium hypochlorite solution was acidified (S1 Fig). In this experiment, before sulfuric acid was added to Sodium hypochlorite, there was a strong peak at 292 nm, but the peak intensity gradually decreased and the intensity of the peak at 236 nm increased. Furthermore, based on the previous discussion, it is thought that sodium hypochlorite (292 nm) changes to hypochlorous acid (236 nm) upon acidification. On the other hand, it is possible that the sodium chlorite solution is less likely to be converted to chlorous acid. As with sodium hypochlorite, the chlorite IC peak should not change with pH. However, according to our experimental results, the amount of chlorite ions gradually decreased as the solution was made acidic. This indicates the possibility that sodium chlorite does not change to chlorous acid even if it becomes acidic. As can be seen from the isosbestic point, chlorous acid may have changed directly to a species that gives an absorption peak at 365 nm.

Acidic sodium chlorite solution, ASC [11], is permitted by the Food and Drug Administration of the United States and is widely used to disinfect many kinds of foods [9,10]. This experiment in which sulfuric acid was added to sodium chlorite solution may have reconfirmed the synthetic method for ASC solution. A small fluctuation was observed in the peak at 365 nm, which may be due to the presence of unstable species. Further investigation is needed to identify the species absorbing at 365 nm.

### Identity of species exhibiting maximum absorption at 365 nm in acidic sodium chlorite solution

**Fig 6:**
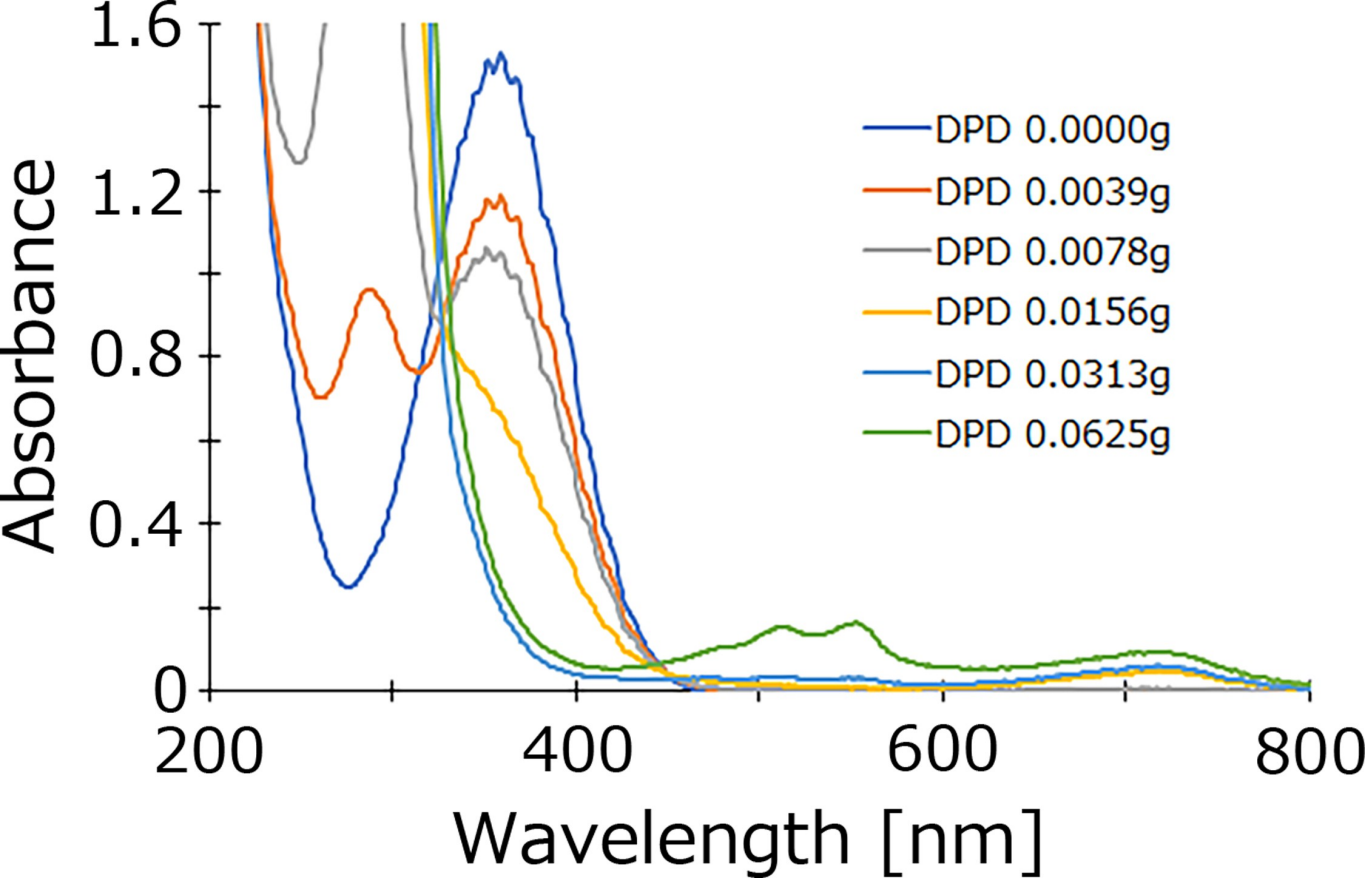
Absorption spectra of acidic chlorite solutions with different concentrations of DPD reagents. Measured absorption spectra from 200 to 800 nm. 32 mM sodium chlorite solutions and equal volume of 500 mM sulfuric acid were mixed and were measured. The solution was incubated for 100 min before analysis, and at least three independent experiments were performed.

Based on ESR experiments, Kawada et al. proposed that the species that exhibits maximum absorption at 365 nm is the chlorine peroxide radical [14], which is consistent with the results of our experiment. Sodium chlorite solution cannot be measured by the DPD method, but sodium chlorite acidified with sulfuric acid can be measured (Fig 4). The DPD method measures the amount of species with oxidizing power. This suggests the possibility that such species are generated from sodium chlorite acidified with sulfuric acid. As the acidity increases, the bactericidal activity becomes stronger. Thus, the species with maximum absorption at 365 nm may be the species with bactericidal activity [13,22]. In our experiments, we added the DPD reagent to the acidified sodium chlorite solution. Although there was a strong peak at 365 nm before the addition, the peak intensity gradually decreased and that of the peak at 510 nm increased (Fig 6). The increase in the 510 nm peak upon reaction with the DPD reagent indicates that species with maximum absorption at 365 nm have oxidative power. It is common for oxidizing species to be radicals such as superoxides. Sodium hypochlorite and sodium chlorite do not undergo similar reactions, and sodium chlorite may have a completely different reaction mechanism from sodium hypochlorite when it changes from alkaline to acidic. It is very interesting that a trace amount of chlorate ions was detected by IC. Further investigation is required to clarify what species exhibit maximum absorption at 365 nm, and whether they are radicals.

### Is ion chromatography effective for elucidating composition of sodium chlorite solutions?

To clarify the compositions of chlorine-based compounds in acidic sodium chlorite solution, various acidic sodium chlorite solutions were evaluated using IC (Fig 5). Although the chloride ion peak did not change upon acidification, the chlorite ion peak gradually decreased in intensity. This is a result that can only be obtained with IC. The reduction of the sodium chlorite solution determined by IC was similar to that of sodium chlorite and acidic sodium chlorite solutions determined by absorption photometry experiments. Interestingly, the amount of chlorate ion increased slightly with acidity. After IC, no peak at 365 nm was observed by a UV detector connected in series (Fig 5B). Acidified sodium chlorite solutions usually show a large peak at 365 nm. Furthermore, when the chloric acid standard solution was measured with a spectrophotometry, no absorption spectrum appeared at 365 nm (S2 Fig). This result suggests the possibility that the species giving a peak at 365 nm in the sodium chlorite solution changed to other ions that could not be detected by IC, or changed to another chlorinated compound that could not be detected under our measurement conditions. The reason for this is unknown, but there is a possibility that other species besides chlorous acid, which have been proposed in the past, are produced in an acidic sodium chlorite solution with sulfuric acid. Our research introduces new techniques for elucidating the composition of sodium chlorite solutions by adding IC to conventional methods. Admittedly, our IC system has the drawback of not being able to measure positive ions. Since the analysis is for sodium chlorite and sodium hypochlorite, the presence of cations such as sodium ions is also important. Anyway, it is the first step to analyze by combining IC and other analysis equipment. To shed light on this problem, it will be necessary to perform additional studies using different IC conditions and eluent compositions.

## Conclusions

In this study, we attempted to identify species present in sodium chlorite solution. It was found that the composition is very different in acidic and alkaline solutions. However, the exact identity of the species is unknown at this stage. Further investigation is needed to determine the composition of acidic chlorite acid, including the content of chlorine peroxide radicals. There is no doubt that the species giving an absorption peak at 365 nm is the main component in the acidic sodium chlorite solution. Moreover, sodium chlorite may have converted directly into radicals. IC can be used to reveal the true ionic composition of small samples. The conventional iodometric titration (KI) and DPD methods can only measure total available chlorine and free available chlorine volumes. In addition, it is difficult to gauge the accuracy because titration is performed manually. An ESR spectrometer is a very good measuring instrument, but it is expensive and requires specialized skills. It is necessary to continue to develop an accurate quantitative separation method using IC for the active ingredients of sodium chlorite solution. In particular, the relationship between the degree of bactericidal activity and the amount of the species absorbing at 365 nm in antibacterial tests, and the development of quantitative methods for species that can serve as simple indicators of bactericidal activity in IC, require further investigation.

## Supporting information

S1 Fig 16 mM NaOCl + different concentration of H2SO4. (XLSX)

S2 Fig 16mM NaClO3 solution + Each concentration of NaClO3. (XLSX)

S1 Data (XLSX)

S2 Data (XLSX)

S3 Data (XLSX)

S4 Data (XLSX)

S5 Data (XLSX)

S6 Data (XLSX)

S7 Data (XLSX)
